# Induced Thermotolerance and Expression of Three Key *Hsp* Genes (*Hsp70*, *Hsp21*, and *sHsp21*) and Their Roles in the High Temperature Tolerance of *Agasicles hygrophila*

**DOI:** 10.3389/fphys.2019.01593

**Published:** 2020-01-14

**Authors:** Jisu Jin, Meiting Zhao, Yao Wang, Zhongshi Zhou, FangHao Wan, Jianying Guo

**Affiliations:** ^1^State Key Laboratory for Biology of Plant Diseases and Insect Pests, Institute of Plant Protection, Chinese Academy of Agricultural Sciences, Beijing, China; ^2^College of Plant Protection, Hunan Agricultural University, Changsha, China; ^3^College of Agriculture, Ludong University, Yantai, China

**Keywords:** *Agasicles hygrophila*, *Alternanthera philoxeroides*, heat shock protein (Hsp), RT-qPCR, RNAi, fecundity, survival

## Abstract

Thermal adaptation plays a fundamental role in the expansion and distribution of insects, and heat shock proteins (Hsps) play important roles in the temperature adaptation of various organisms. To determine the roles of Hsp genes (*Hsp70*, *Hsp21*, and *sHsp21*) on the high temperature tolerance of *Agasicles hygrophila*, we obtained complete cDNA (complementary DNA) sequences for *Hsp70*, *Hsp21*, and *sHsp21* by rapid amplification of cDNA ends (RACE), analyzed their expression profiles under different high temperature treatments by quantitative reverse transcription polymerase chain reaction (RT-qPCR), and performed functional verification by RNA interference (RNAi). The open reading frames of *Hsp70*, *Hsp21*, and *sHsp21* were 1940, 543, and 567 bp, encoding 650, 180, and 188 amino acids, respectively. Their molecular weights (MWs) were 71.757, 20.879, and 21.510 kDa, and the isoelectric points were 5.63, 6.45, and 6.24, respectively. Phylogenetic tree analysis showed that the *Hsp70*, *Hsp21*, and *sHsp21* genes of *A. hygrophila* were relatively conserved in evolution. The *Hsp70* and *Hsp21* genes in *A. hygrophila* were homologous to those in *Leptinotarsa decemlineata* (87 and 79% similarity, respectively), and the *sHsp21* gene in *A. hygrophila* was homologous to that in *Lissorhoptrus oryzophilus* (74% similarity). The amino acid polypeptide chain had highly conserved sequences of DLGGGTFD, VLVGGSTR, and GPTIEEVD. The sequence of EEVD was the characteristic motif of cytoplasmic *Hsp70*, and the highly conserved sequences of MALFR and MSLLP were characteristic sequences of *Hsp2* and *sHsp21*, respectively. Relative quantitative real time PCR showed that the three *Hsps* could be induced by 4-h treatment at high temperatures. Significant upregulation of these *Hsps* was observed when the temperature was further increased. The RNAi results showed that the injection of the three *Hsps*’ dsRNA could suppress the expression at the gene level significantly. Compared with the control group, high temperature heat shock reduced the fecundity of *A. hygrophila* significantly, and the fecundity decreased with the increase in temperature. Our results suggest that *Hsp70*, *Hsp21*, and *sHsp21* might play key roles in high temperature adaptation of *A. hygrophila* and help improve our understanding of their mechanism of thermotolerance.

## Introduction

Alligator weed *Alternanthera philoxeroides* (Mart) Griesb (Amaranthaceae) is an aquatic and clonal weedy species native to South America (Buckingham, 1996). It has been recognized as a prominent problematic weed worldwide and is listed as one of the 16 most serious invasive species in China (State Environmental Protection Administration of China, and Chinese Academy of Sciences, 2003). *Alternanthera philoxeroides* was firstly introduced into China in the 1930s as a forage crop (Wang et al., 2005), and presently, it occurs in most regions of southern China and has become one of the most noxious weeds in China. Some invasive plants reach high densities and cause economic or environmental harm or threaten human health (National Invasive Species Council, 2001). The infestation of *A. philoxeroides* creates mats that restrict traffic in navigable waterways and limit fishing, swimming, and other recreational uses of lakes, rivers, and streams (Spencer and Coulson, 1976). The infestation also destroys landscape and biological diversity and threatens ecological integrity of the environment (Vogt et al., 1992; Ma and Wang, 2005). The alligator weed is a well-adapted species and it can reproduce both sexually and asexually, although the seeds are often not viable (Julien et al., 1995).

Biological control is a powerful management method with the potential to control invasive weeds without having to resort to expensive and temporary mechanical or chemical methods (Ma et al., 2010). Insects have been used successfully as biological control agents to suppress alligator weed in Florida and other states of the southeastern United States (Gangstad, 1976). The alligator weed flea beetle *Agasicles hygrophila* Selman & Vogt was the first host-specific insect introduced into the United States to control alligator weed in 1964 (Zeiger, 1967; Spencer and Coulson, 1976). It was first introduced into China from Florida in the United States to control alligator weed in 1986, and has been successfully used in southern China (Wang, 1989; Ma et al., 2003).

Temperature affects the distribution and abundance of living organisms, and is particularly important for ectotherms such as insects (Yu et al., 2012). According to our field survey in Hunan province, the population of *A. hygrophila* has two obvious peaks from June to July and October to November, but it drops sharply from August to September, leading to inefficient control of alligator weed (Li et al., 2011). Studies have shown that heat shock proteins (Hsps) exist widely in animals and likely play important roles in their thermal adaptation and thermo-tolerance or thermo-protection (Feder and Hofmann, 1999; Hoffmann et al., 2003; Fangue et al., 2006; MacRae, 2010; Lü et al., 2014). Under heat shock stress, the heat shock transcription factor (HSF) drives the expression of a broad range of heat-responsive genes, including *Hsp70* and *Hsp90*, which are also expressed during the development of insects under normal conditions (Westwood et al., 1991). Different classes of Hsps can play different roles in the thermotolerance of certain species (Goto et al., 1998; Rinehart et al., 2000, 2007; Goto and Kimura, 2004; Aruda et al., 2011). To date, the characteristics of *Hsp* genes and their mRNA expression profiles in *A. hygrophila*, as well as the relationship between *Hsp* gene expression and thermotolerance, remain unknown.

To reveal the potential function of different *Hsp* genes in the thermotolerance of *A. hygrophila*, we isolated complete cDNA (complementary DNA) sequences for *Hsp70*, *Hsp21*, and *sHsp21* by rapid amplification of cDNA ends polymerase chain reaction (RACE-PCR), and examined the expression patterns of these *Hsp* genes under different high temperature treatments. We also analyzed the phylogenetic relationships of these three *Hsps* by comparing them with *Hsps* from other insects. In addition, we used an RNA interference technique to examine their roles in heat tolerance. The present results could improve our understanding of the mechanisms of thermotolerance in *A. hygrophila* at the molecular level.

## Materials and Methods

### Experimental Insects and Plants

*Agasicles hygrophila* adults were collected from a pond field covered with alligator weed in Changsha, Hunan province, China (28°11′49′′ N, 112°58′42′′ E) in June 2016 by the sweeping method and were maintained on alligator weed plants in the laboratory at the Chinese Academy of Agricultural Sciences, Beijing, China (BJ, CAAS) under controlled conditions, which were 26 ± 2°C, 75 ± 5% relative humidity (RH), and a 12:12 h light–dark regime. The flea beetles were cultivated for three generations to eliminate maternal effects before the experiments.

Alligator weeds were collected from a pond at the Institute of Plant Protection, Hunan Academy of Agricultural Sciences and planted in sterilized soil in plastic boxes (30 cm × 30 cm × 15 cm) with sterilized soil. Alligator weeds were planted in the soil in a greenhouse at Langfang Experimental Station, Chinese Academy of Agricultural Sciences (LF, CAAS), Hebei province and watered daily.

### Temperature Processing and Sample Collection

Newly emerged *A. hygrophila* adult beetles, which were less than 12 h old after eclosing were used for the experiments. Ten females and 10 males were put together in cylindrical boxes of 8 cm in diameter and 12 cm in height. Each temperature was studied using 10 boxes of *A. hygrophila* adult beetles. These beetles were exposed to heat shock at 30, 36, or 39°C for 4 h from 10:00 to 14:00 in a constant temperature incubator (RPX-450, Colin, Beijing) daily. The temperatures used were based on the temperature ranges in summer in Changsha, Hunan province of China (Zhao, 2017). Five females and 5 males were collected from each temperature group every day for a week. Each sample with 10 beetles was put into a 1.5 ml microtube, frozen in liquid nitrogen immediately, and subsequently kept in the refrigerator (DW-86L628, Haier Special Electric Appliance, Co., Ltd., Tsingtao) at −80 ± 1°C. The beetles at 30°C were used as the control group, and the heat-shock test and sample collection were replicated three times for each temperature treatment.

### RNA Extraction and cDNA Synthesis

Total RNA from the samples was extracted using the TRIzol (Trizol reagent 10296028, United States) method and the reverse transcription kit (TransScript One-Step gDNA Removal and cDNA Synthesis SuperMix, AT311-03, TransGen Biotech, China) was used to synthesize first-strand cDNA and remove genomic DNA. Total RNA was stored in a −80 ± 1°C refrigerator prior to subsequent experimentation.

### Cloning the Full-Length cDNA of *Hsps*

Specific primers were designed according to the cDNA obtained in step 2.3; then, 5′/3′ rapid amplification of cDNA ends (RACE) was performed to obtain full-length cDNAs according to the manufacturer’s user manual (SMARTer^®^ RACE 5′/3′, Cat. No(s). 634858, Clontech Laboratories, Inc., Takara, CA, United States) using gene-specific primers corresponding to GSP1 and GSP2 (Supplementary Table S1). To ensure that the 5′ and 3′ fragments were derived from the same gene, specific primer sets flanking the open reading frames (ORFs) were designed and used to amplify the full-length cDNAs.

### Sequence Analysis of *Hsp* cDNA

The full-length cDNAs of *Hsps* were used as query sequences to search for other insect *Hsps* in GenBank by BLAST software available at the NCBI website^1^. Sequence alignment and identity analyses were carried out with DNAMAN and MEGA6 or vector NTI. ORF Finder^2^ was used to find the ORF of cDNA. The amino acid sequences and the MW of the proteins were calculated using DNASTAR.

### Homologous Alignment and Phylogenetic Analysis

To evaluate the molecular evolutionary relationship of *Hsps* in *A. hygrophila* with those in other insects, phylogenetic trees were constructed based on their amino acids sequences by MEGA 6 version 5 using the Neighbor-Joining (NJ) method (Tamura et al., 2011).

### Relative Quantitative Real Time PCR

The PCR reactions were performed in a 20 μl total reaction volume including 10 μl of 2 × TransStart^®^ Tip Green qPCR SuperMix (TransGen Biotech, China), 0.4 μl of Passive Reference Dye II, 0.4 μl of each of the gene-specific primers, 1 μl of cDNA templates, and 7.8 μl of ddH_2_O. Real-time PCR was carried out with specific primers to determine the expression level of *Hsp70*, *Hsp21*, and *sHsp21*. β*-actin* was used as an internal reference gene. Reactions were carried out on the 7500 Real-Time PCR System (Applied Biosystems, Grand Island, NY, United States). The thermal cycler parameters were as follows: 94°C for 30 s followed by 40 cycles each at 94°C for 5 s, and 60°C for 34 s. At the end was the melting curve stage following the default parameters. A standard curve was derived from the serial dilutions to quantify the copy numbers of target mRNAs. The standard curves for *Hsp70*, *Hsp21*, *sHsp21*, and the β*-actin* had slopes of −3.214, −3.313, −3.325, and −3.415; correlation coefficients (*R*^2^) of 0.998, 0.994, 0.991, and 1.000; amplification efficiencies of 104.714, 100.361, 99.884, and 96.265; and Y-intercepts of 44.530, 44.201, 47.418, and 49.371, respectively. Each cDNA sample was assayed in triplicate. The data were analyzed based on the Cp method according to the mathematical model of Pfaffl (Fleige et al., 2006), simplified to 2^–△△Ct^ as follows:

Ct△△=(Cptarget-Cpreference)-treatment

(1) (Cptarget-Cpreference).control

### Synthesis of Double-Stranded RNA

Three *Hsp* transcription templates were produced from the total cDNA of *A. hygrophila* using gene-specific primers containing a T7 promoter sequence; the T7 promoter was as described by Ghanim et al. (2007). The promoter sequences were as follows: TAATACGACTCACTATAGGG. Amplification reactions were conducted in 36.5 μl ddH_2_O, 5 μl 10 × PCR buffer (10 μM), 1 μl dNTPs (2.5 mM), 1 μl Taq DNA polymerase (TransGen Biotech, China), 2 μl T7 reverse primer, 2 μl T7 forward primer, and 2.5 μl cDNA template in a final volume of 50 μl. The PCR reaction procedure was 94°C for 5 min, 35 cycles of 94°C for 30 s, 55°C for 30 s, and 72°C for 1 min, and a final extension step of 72°C for 10 min. Amplification of PCR products was confirmed by separation on 1% agarose gels and visualized by staining with GelRed under UV light. The PCR products were purified using an AxyPrepTM DNA Gel Extraction Kit (Axygen) according to the manufacturer’s instructions. The PCR products were stored at −80 ± 1°C prior to the synthesis of dsRNA.

dsRNA was synthesized using the MEGAscript^®^ T7 Transcription Kit (Ambion, Austin, TX, United States), and 1 μg PCR product (plasmid) was used as the transcription template. It was analyzed by 1% agarose gel electrophoresis and quantified spectrophotometrically. The dsRNA was stored at −80 ± 1°C or used for subsequent experiments immediately.

### Expression Analysis of Three *Hsps* After RNAi

Newly emerged adult female beetles (<12 h after eclosing) were collected for dsRNA injection utilizing a PLI-100 Pico-Injector (Harvard Apparatus, Holliston, MA, United States) with an MP-255 Micromanipulator (Sutter, Novato, CA, United States) under an Olympus stereomicroscope. The dsRNA solution (the final amount of dsRNA reached 1.0 μg) was injected into the penultimate 2–3 conjunctivum on the abdomen of *A. hygrophila* female adults. Each experiment was repeated at least three times. Each repeat included 100 female individuals. After injection, every 10 females and 10 freshly emerged adult males were put together in cylindrical boxes of 8 cm in diameter and 12 cm in height with fresh *A. philoxeroides* stems. These beetles were exposed to heat shock at 30, 36, or 39°C for 4 h in a constant temperature incubator (RPX-450, Colin, Beijing), and the climate chamber was maintained at 75 ± 5% RH with a 12:12 h light–dark photoperiod (Guo et al., 2011). Five females were collected and used for expression analysis of the three *Hsp* genes at each temperature every day for a week. The blank control was injected with dsRNAs of *EGFP*.

### Effects of RNAi on Fecundity of *A. hygrophila*

To confirm the specificity of RNAi, dsRNA solution (the final amount of dsRNA reached 1.0 μg) for *Hsp70*, *Hsp21*, and *sHsp21* was injected into insects in the penultimate abdomen of newly emerged adult female beetles. Each treatment included 15 female beetles. Based on the results of the preliminary experiment, we injected about 30 females for our experiment to ensure the survival of 15 females for later experimental observation after injection. One female and one freshly emerged adult male were put together on damp filter paper in a 9 cm diameter Petri dish with fresh *A. philoxeroides* stems and they were exposed to heat shock at 30, 36, or 39°C for 4 h in a constant temperature incubator (RPX-450, Colin, Beijing), and the climate chamber was maintained at 75 ± 5% RH with a 12:12 h light–dark photoperiod (Guo et al., 2011). Each pair of *A. hygrophila* adults was observed, and laid eggs were counted and collected every 24 h for 6 days. The eggs were continuously examined every day and hatching larvae were removed and counted after egg batches began to hatch until no eggs had hatched for one successive week. Each treatment contained 300 eggs, and three replicates were performed for each treatment.

### Adult Survival of *A. hygrophila* After RNAi

Bowler and Terblanche (2008) observed that adults of different ages respond differently to temperature exposure; thus, newly emerged adult *A. hygrophila* females and males (<12 h after eclosing) were collected for dsRNA injection. At least 15 females and 15 males of each treatment were assayed. After injection, one female and one male were put together on damp filter paper in a 9 cm diameter Petri dish with fresh *A. philoxeroides* stems and heat shocked for 4 h at each of the three temperatures (30, 36, and 39°C) in a constant temperature incubator (RPX-450, Colin, Beijing) and the climate chamber was maintained at 75 ± 5% RH, with a 12:12 h light–dark photoperiod (Guo et al., 2011). The survival of female and male adults was recorded daily until death. The blank control was injected with ds*EGFP*.

### Statistical Analysis

Statistical analyses were conducted using the SAS System for Windows V8. One-way analysis of variance (ANOVA; SAS Institute Inc., 1996, United States) was used to analyze the differences among treatments followed by a least significant difference (LSD) test for multiple comparisons. Experimental data were checked for normality and homoscedasticity, and if needed, were arcsine square-root or log-transformed before analysis. Differences among mean values were determined using a LSD test at *P* < 0.05.

## Results

The full-length cDNAs of *Hsp70*, *Hsp21*, and *sHsp21* in *A. hygrophila* were obtained by RT-PCR and RACE-PCR and submitted to NCBI (GenBank accession numbers: MN138034, MN163037, and MN163038, respectively). They contained 1940, 543, and 567 bp, with ORFs encoding 650, 180, and 188 amino acids, respectively (Supplementary Figures S1, S3, S5). Their MWs were 71.757, 20.879, and 21.510 kDa and the oretical isoelectric points were 5.63, 6.45, and 6.24, respectively.

### Sequence Analysis of *Hsp70*

Referring to the DNAMAN multiple alignment, the sequence of *Hsp70* in *A. hygrophila* was highly similar to the *Hsp70* family amino acid sequence of the related insect species (Supplementary Figure S2).

**FIGURE 1:**
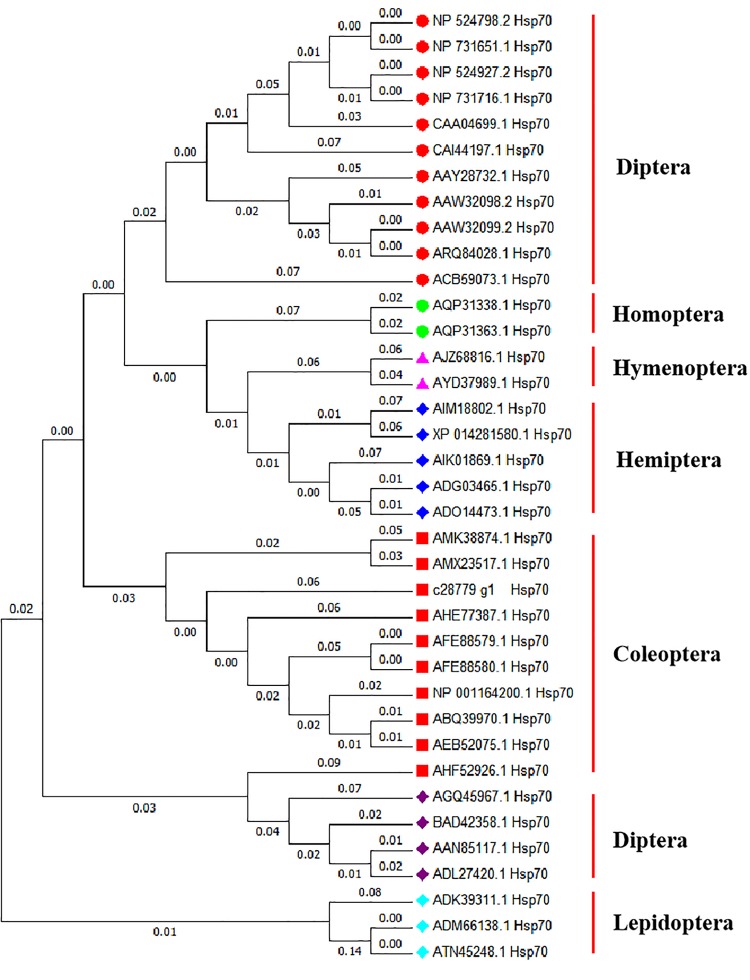
Evolutionary relationships of *Hsp70* based on 37 amino acid sequences. The evolutionary history was inferred using the Neighbor-Joining method (Saitou and Nei, 1987; Zhang and Sun, 2008). The evolutionary distances were computed using the Poisson correction method (Zuckerkandl and Pauling, 1965) and are in the units of the number of amino acid substitutions per site. Evolutionary analyses were conducted in MEGA X (Kumar et al., 2018). *Drosophila melanogaster hsp70Ab* (NP 524798.2), *Drosophila melanogaster hsp70Aa* (NP 731651.1), *Drosophila melanogaster hsp70Bb* (NP 524927.2), *Drosophila melanogaster hsp70Ba* (NP 731716.1), *Drosophila auraria hsp70* (CAA04699.1), *Bactrocera oleae hsp70* (CAI44197.1), *Delia antiqua hsp70* (AAY28732.1), *Liriomyza huidobrensis hsp70* (AAW32098.2), *Liriomyza sativae hsp70* (AAW32099.2), *Liriomyza trifolii hsp70* (ARQ84028.1), *Stratiomys singularior hsp70* (ACB59073.1), *Laodelphax striatellus hsp70* (AQP31338.1), *Nilaparvata lugens hsp70* (AQP31363.1), *Cotesia chilonis hsp70* (AJZ68816.1), *Trichogramma chilonis hsp70* (AYD37989.1), *Bemisia tabaci hsp70* (ADG03465.1), *Bemisia tabaci hsp70* (ADO14473.1), *Orius sauteri hsp70* (AIK01869.1), *Empoasca onukii hsp70* (AIM18802.1), *Halyomorpha halys hsp70* (XP 014281580.1), *Colaphellus bowringi hsp70* (AMK38874.1), *Monochamus alternatus hsp70* (AMX23517.1), *Lissorhoptrus oryzophilus hsp70* (AHE77387.1), *Tenebrio molitor hsp70* (AFE88579.1), *Tenebrio molitor hsp70* (AFE88580.1), *Tribolium castaneum hsp70* (NP 001164200.1), *Anatolica polita borealis hsp70* (ABQ39970.1), *Microdera punctipennis hsp70* (AEB52075.1), *Colaphellus bowringi hsp70* (AHF52926.1), *Diamesa cinerella hsp70* (AGQ45967.1), *Chironomus yoshimatsui hsp70* (BAD42358.1), *Chironomus tentans hsp70* (AAN85117.1), *Chironomus riparius hsp70* (ADL27420.1), *Plutella xylostella hsp70* (ADK39311.1), *Spodoptera litura hsp70* (ADM66138.1), *Mythimna separata hsp70* (ATN45248.1).

Homology analysis revealed that the amino acid sequence derived from the *Hsp70* gene of *A. hygrophila* is highly similar to that encoded by homologous genes of other insect species, among which the similarity to the potato beetle was the highest (*Leptinotarsa decemlineata*) (Figure 1). The amino acid sequences derived from *Hsp70* were highly conserved among insects.

*Hsp70* sequences from Coleoptera were clustered in one branch, indicating that these *Hsp70* sequences had relatively similar amino acid sequences. The *Hsp70* sequence of *A. hygrophila* is most closely related to that of *Leptinotarsa decemlineata* (Figure 1).

### Sequence Analysis of *Hsp21*

**FIGURE 2:**
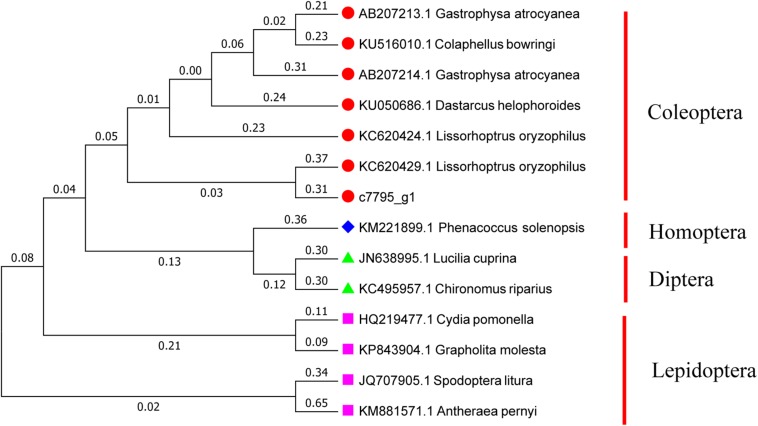
Gene system development of the Neighbor-Joining phylogenetic tree of *Hsp21* in *A. hygrophila*. This tree is used to determine the relationships between different insects. The numbers above the branches indicate the percentages of bootstrap replicates in which each species is grouped together. The scale bar indicates the number of substitutions per site for each unit branch length. *Colaphellus bowringi* (AMK38872.1), *Gastrophysa atrocyanea* (BAD91164.1), *Lissorhoptrus oryzophilus* (AHE77375.1), *Agasicles hygrophila* (AHH25011.1), *Dastarcus helophoroides* (ANA11235.1), *Harmonia axyridis* (ART30112.1), *Harmonia axyridis* (ART30114.1), *Gastrophysa atrocyanea* (BAD91165.1), *Lissorhoptrus oryzophilus* (AHE77378.1), *Lissorhoptrus oryzophilus* (AHE77381.1), *Galeruca daurica* (ATY47553.1), *Tribolium castaneum* (XP_973344.1), *Tribolium castaneum* (XP_973378.1), *Macrocentrus cingulum* (ACF21815.1), *Liriomyza huidobrensis* (ABE57137.1), *Musca domestica* (AHK23446.1), *Galleria mellonella* (AXY94831.1), *Spodoptera litura* (ADK55523.1), *Helicoverpa armigera* (ATB54991.1), *Bicyclus anynana* (AFM73645.1), *Eogystia hippophaecolus* (AYA93247.1), *Pteromalus puparum* (ACO57620.1), *Pteromalus puparum* (AEM45800.1), *Helicoverpa armigera* (ATB54994.1), *Bombyx mori* (NP_001036985.1).

Referring to the DNAMAN multiple alignment, the sequence of *Hsp21* in *A. hygrophila* was highly similar to the *Hsp21* family amino acid sequence of the related insect species (Supplementary Figure S4). Homology and phylogenetic tree analysis revealed that the amino acid sequence derived from the *Hsp21* gene of *A. hygrophila* is highly similar to that encoded by homologous genes of other insect species, among which the similarity to *Lissorhoptrus oryzophilus* was the highest (Figure 2).

### Sequence Analysis of *sHsp21*

**FIGURE 3:**
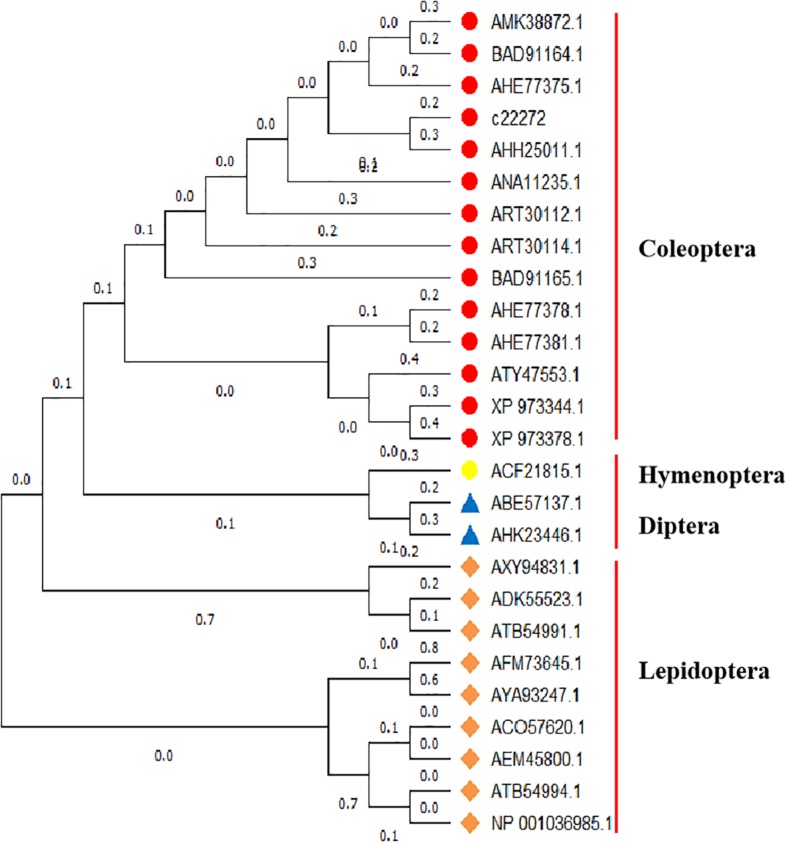
Gene system development of the Neighbor-Joining phylogenetic tree of *sHsp21* in *A. hygrophila*. This analysis involved 28 amino acid sequences, and evolutionary analyses were conducted in MEGA X (Kumar et al., 2018). *Lissorhoptrus oryzophilus* (AHE77375.1), *Colaphellus bowringi* (AMK38872.1), *Agasicles hygrophila* (AHH25011.1), *Harmonia axyridis* (ART30114.1), *Dastarcus helophoroides* (ANA11235.1), *Gastrophysa atrocyanea* (BAD91164.1), *Lissorhoptrus oryzophilus* (AHE77378.1), *Lissorhoptrus oryzophilus* (AHE77381.1), *Choristoneura fumiferana* (ASQ43188.1), *Galeruca daurica* (ATY47553.1), *Harmonia axyridis* (ART30112.1), *Tribolium castaneum* (XP973344.1), *Helicoverpa armigera* (ATB54994.1), *Chilo suppressalis* (AGM90557.1), *Chilo suppressalis* (AGM90555.1), *Liriomyza huidobrensis* (ABE57137.1), *Musca domestica* (AHK23446.1), *Pteromalus puparum* (AEM45800.1), *Gastrophysa atrocyanea* (BAD91164.1), *Fenneropenaeus chinensis* (AEE81035.1), *Spodoptera litura* (ADK55523.1), *Helicoverpa armigera* (ATB54991.1), *Galleria mellonella* (AXY9483.1), *Eogystia hippophaecolus* (AYA93248.1), *Eogystia hippophaecolus* (AYA93247.1), *Bicyclus anynana* (AFM73645.1), *Tribolium castaneum* (XP973378.1), *Lissorhoptrus oryzophilus* (AHE77382.1), *Gastrophysa atrocyanea* (BAD91165.1).

Referring to the DNAMAN multiple alignment, the sequence of *sHsp21* in *A. hygrophila* was highly similar to the *sHsp21* family amino acid sequence of the related insect species (Supplementary Figure S6). Homology and phylogenetic tree analysis revealed that the amino acid sequence derived from the *sHsp21* gene of *A. hygrophila* is highly similar to that encoded by homologous genes of other insect species, among which the similarity to *Lissorhoptrus oryzophilus* was the highest (Figure 3).

### Expression of *Hsp70*, *Hsp21*, and *sHsp21* Under Thermal Stress

**FIGURE 4:**
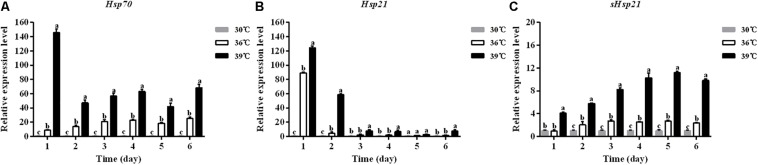
The mRNA expression profiles of three *Hsp* genes in *A. hygrophila*. The relative levels of *Hsp* mRNAs were examined at different temperatures (30, 36, and 39°C). Values are the means ± SD. The data were analyzed by one-way ANOVA followed by the least significant difference (LSD) test. Different letters indicate significant differences between means (*P* < 0.05). **(A)** Expression level of *Hsp70*, **(B)** the expression level of *Hsp21*, and **(C)** expression level of *sHsp21*.

The expression levels of three *Hsp* mRNAs in *A. hygrophila* under standard (30°C) and high (36, 39°C) temperature treatments were determined by relative quantitative real time PCR. The expression of β*-actin* was used as an internal control. The results revealed that these three *Hsps* could be induced by 4 h treatment at high temperatures (Figure 4). Significant up-regulation of *Hsps* was observed when the temperature was further increased. At 39°C, the expression level of *Hsp70* was significantly higher than that at 30°C (Figure 4A). The *Hsp70* mRNA level was significantly higher at 36°C compared with that at 30°C (Figure 4A).

The expression levels of *Hsp21* in *A. hygrophila* were significantly higher at 39°C than at 30°C during the 6 days of measurement (Figure 4B). Two days after the daily 4 h heat shock treatment, the *Hsp21* mRNA level was significantly higher at 36°C treatment compared with that at 30°C (Figure 4B). However, from days 3 to 6, there was no significant difference in the *Hsp21* mRNA level between 36 and 30°C treatments (Figure 4B).

The expression levels of *sHsp21* in *A. hygrophila* were significantly higher at 39°C than those at 30°C during the 6 days of measurement (Figure 4C). One day after the daily 4 h heat shock treatment, there was no significant difference in the *sHsp21* mRNA level between 36 and 30°C treatments. However, from days 2 to 6, the *sHsp21* mRNA level was significantly higher after 36°C treatment than that after 30°C treatment (Figure 4C).

### Effects of dsRNA Injection on Expression of Three *Hsps*

**FIGURE 5:**
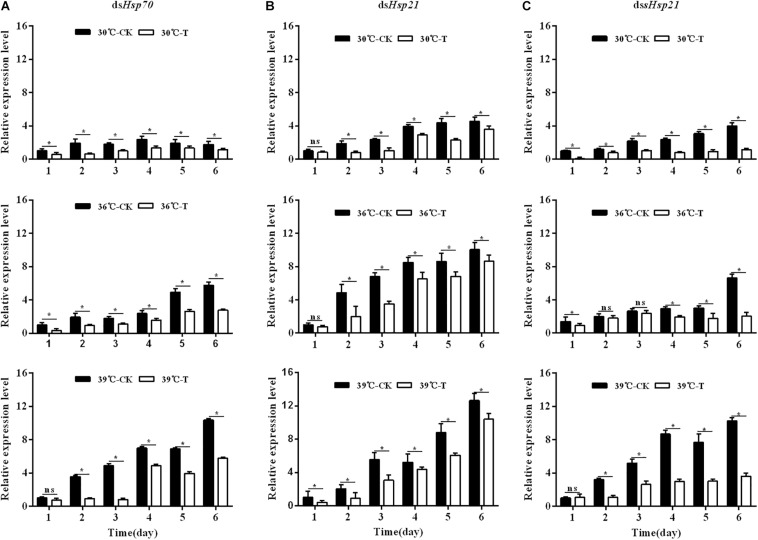
Relative expression levels of *Hsp70*, *Hsp21*, and *sHsp21* after injection of dsRNA into freshly emerged *A. hygrophila* adults (**A,** *Hsp70*; **B**, *Hsp21*; **C**, *sHsp21*). Values are means ± SD. The data were analyzed by Student’s *t*-test. ^∗^*P* < 0.05. ns, not significant.

Injection of ds*Hsp70*, ds*Hsp21*, and ds*sHsp21* into freshly emerged *A. hygrophila* adults could significantly inhibit the endogenous expression of *Hsp* mRNAs (Figure 5). From days 2 to 6 after the injection of ds*Hsp70*, ds*Hsp21*, and ds*sHsp21*, the expression levels of these genes decreased significantly compared with the control group with the injection of ds*EGFP* (Figure 5). The expression levels of mRNAs were up-regulated significantly after heat shock at 36 and 39°C for 4 h every day, while the injection of dsRNAs suppressed the expression at the gene level (Figure 5).

### Effects of RNAi on *A. hygrophila* Fecundity

**FIGURE 6:**
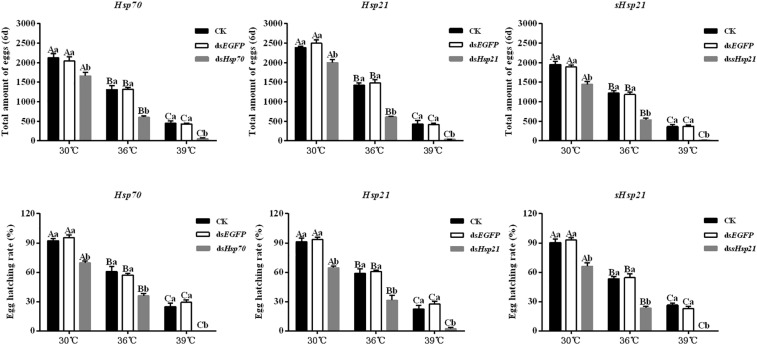
Fecundities and egg hatching rate of *A. hygrophila* after injection of *dsHsp70*, *dsHsp21*, and *dssHsp21*. Different amounts of *dsEGFP* were injected as the positive control. CK represents the no treatment as the negative control. Capital letters indicate the difference of the egg hatching rate and fecundity capacities of the *A. hygrophila* at the different temperatures. Lowercase letters refer to the differences in the egg hatching rate and fecundity capacities with different treatment of the same temperature. All values are shown as the mean ± SD. The data were analyzed by one-way ANOVA followed by the least significant difference (LSD) test. Different letters indicate significant differences between means (*P* < 0.05).

**FIGURE 7:**
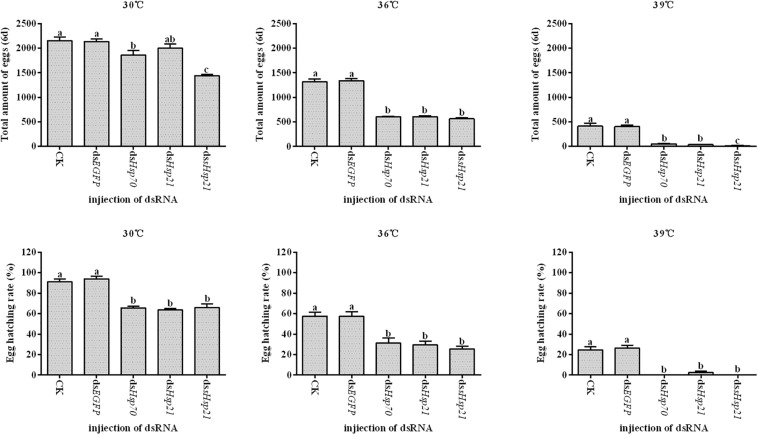
Fecundities and Egg hatch rate of *A. hygrophila* after injection of ds*Hsp70*, ds*Hsp21*, and ds*sHsp21.* All values are shown as the mean ± SD. The data were analyzed by one-way ANOVA followed by the least significant difference (LSD) test. Different letters indicate significant differences between means (*P* < 0.05).

**FIGURE 8:**
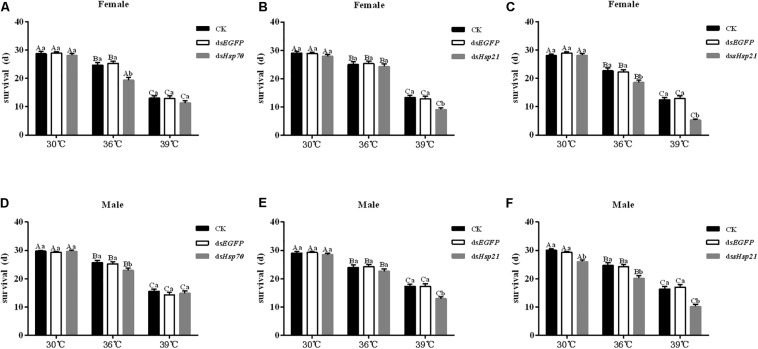
Adult survival results of *A. hygrophila* after injection of ds*Hsp70*, ds*Hsp21*, and ds*sHsp21*. Different amounts of ds*EGFP* were injected the positive control. CK represents the no treatment as the negative control. Capital letters indicate the difference of the survival rates of the *A. hygrophila* at the different temperatures of 30, 36, and 39°C. All values are shown as the mean ± SE. The data were analyzed by one-way ANOVA followed by the least significant difference (LSD) test.

After the injection of ds*Hsp70*, ds*Hsp21*, or ds*sHsp21* into the freshly emerged *A. hygrophila* female abdomen, the fecundity of the female beetle was significantly lower than that of the control group with the injection of ds*EGFP*. The decrease was more dramatic with the increasing heat-shock treatment temperatures. At 30°C, there were no significant differences between the treatment and control groups. However, heat shock at 36 or 39°C led to a more significant decrease in the fecundity of *A. hygrophila* than treatment at 30°C (Figure 6). Figure 7 shows the differences between the three *Hsp* genes at different temperatures. At 30 and 39°C, the number of laid eggs for the ds*shsp21* group was significantly lower than that of the ds*Hsp70* and ds*Hsp21* group. While the number of laid eggs for the ds*Hsp70* group was not significantly difference compared with the ds*Hsp21* group. At 36°C, there were no difference in laid eggs between three *Hsp* genes. Additionally, there were no difference in egg hating rate between three *Hsp* genes at the different temperatures (Figure 7). The silencing of these three *Hsps* not only significantly affected the reproduction of *A. hygrophila* but it also depressed the survival of *A. hygrophila* significantly (Figure 8).

## Discussion

Hsps are relatively conserved in evolution and play important roles in protecting living organisms from damage. Under physiological stress conditions, Hsps can maintain the normal structure of proteins and maintain the stability of the cytoskeleton (Tissières et al., 1974; Salvucci et al., 2000).

Studies have shown that Hsps, as important stress proteins, play a very important role in the stress resistance of organisms, and the *Hsp* gene may be one of the genes most closely related to temperature stress (Dahlgaard et al., 1998).

Previous studies on various *Hsp* genes have used cloning in insects, such as in *Delia antiqua* (Bin et al., 2006), *Tribolium castaneum* (Mahroof et al., 2005), *Cotesia vestalis* (Shi et al., 2013), and *Microdera punctipennis* (Ou et al., 2014). In this study, we cloned a full-length cDNA of 2053 bp encoding *Hsp70* in *A. hygrophila*. The length of the ORF was 1940 bp, encoding 646 amino acids. The amino acid sequence of *Hsp70* has a typical cytoplasmic characteristic motif EEVD (Demand et al., 1998; Vayssier et al., 1999). In addition, this sequence contains a GPTIEEVD sequence, which is included in most organisms, indicating that this regulatory sequence is highly conserved (Su et al., 2010). Moreover, based on the intermediate fragment, the 5′ RACE and 3′ RACE specific primers of *Hsp21* and *sHsp21* were designed, and the *Hsp21* and *sHsp21* sequences with lengths of 549 and 693 bp, respectively, were finally obtained. The ORFs of *Hsp21* and *sHsp21* were 538 and 567 bp, encoding 180 and 188 amino acids, respectively. Sequence analysis revealed that Hsp21 and sHsp21 were members of the small Hsp family including conserved domains such as the n-amino acid terminal domains, the -crystallin domains, and the c-amino acid terminal domains (Jong et al., 1998). Phylogenetic tree analysis showed that small MW Hsps of *A. hygrophila* and the small MW Hsps of other Coleoptera insects had high homology, so we speculate that they have a long independent evolutionary history.

In our current study, the construction of phylogenetic trees indicated that *Hsp70*, *Hsp21*, and *sHsp21* genes are relatively conserved in evolution. Each tested *Hsp* gene in *A. hygrophila* could be clustered into one branch with other Coleoptera species of the same family, which could be well-distinguished from Hemiptera, Dictyoptera, Lepidoptera, Hymenoptera, and Diptera insects (Lü et al., 2014), indicating that Hsp70, Hsp21, or sHsp21 of Coleoptera insects have relatively similar amino acid sequences. At present, insect small Hsps are an infrequently studied part of the superfamily of Hsps, and the overall classification is not detailed enough. Specifically, the classification of sHsp21 of Coleoptera insects is limited. The research on small Hsps mainly focuses on a few model insects, such as *Drosophila melanogaster* (Tissières et al., 1974) and *Bombyx mori* (Li et al., 2009).

Our study showed that *Hsp70*, *Hsp21*, and *sHsp21* genes in *A. hygrophila* showed similar expression patterns. The expression levels of these three *Hsps* increased with the increasing heat shock temperatures and were all higher than those in the control groups. These results are consistent with other previous studies showing significant up-regulation of *Hsp* gene expression levels when temperature was increased (Lü and Wan, 2011). It has been traditionally accepted that the expression levels of *Hsps* in the examined tissues changed obviously under high temperature treatments (Huang et al., 2007, 2009). However, in the study of *Antheraea pernyi*, the expression of *sHsp21* was not obviously changed in fat bodies under high temperatures (Liu et al., 2013). *Hsp* genes are not only involved in thermal response but also may be related to reproduction in insects. For example, the expression of *sHsp* was also detected during male gametogenesis in *Drosophilia melanogaster* (Joanisse et al., 1998). Although the expression of *Hsp70*, *Hsp21*, and *sHsp21* genes was detected in *A. hygrophila* after heat shock treatment in this study, determining whether these *Hsp* genes play a role in the reproduction of this beetle needs further study.

RNAi technology has been successfully applied to the studies of gene function in insects (Hannon, 2002; Ghanim et al., 2007). There are also successful examples in Coleoptera (Arakane et al., 2005; Shi et al., 2016). In our study, the target genes *Hsp70*, *Hsp21*, and *sHsp21* were silenced with high efficiency in *A. hygrophila*, and the expression of these three *Hsps* was significantly down-regulated after RNA interference. Suppression of these three *Hsps* negatively affected fecundity and adult survival, causing significant declines in egg laying and egg hatch rates. Previous studies have demonstrated that heat shock protein genes affect insect reproduction and longevity. Xie et al. (2019) studied the functional of *hsp18.3* gene in the *Tribolium castaneum* and found that after injection of ds*hsp18.3*, one pair of the beetles can hardly spawn. When Tc*hsp18.3* was knocked down, the survival rate of adults and fecundity were significantly lower than the control group. Lapierre and Hansen (2012), Wiese et al. (2012), and Yanos et al. (2012) explored the lifespan of nematode *Caenorhabditis elegans* and found that the *Hsp* genes could be placed into longevity regulating pathway of *C. elegans* as well as improving the longevity of *C. elegans*. This phenomenon indicates that *Hsp70*, *Hsp21*, and *sHsp21* play important roles both in the reproduction and in the thermo-tolerance of *A. hygrophila.* Additionally, previous studies also found that there was functional compensation between genes. Petko and Lindquist (1986) and Bossier et al. (1989) found yeast may possess (an)other, as yet unidentified, small *Hsp*(s), which could compensate for the lack of *Hsp26* function. Zhang et al. (2019) found that there was strong functional compensation among these three *AhVgs*. In our study, whether these three *Hsp* genes have the functional compensation in regulating thermotolerance in *A. hygrophila* needs to be further investigated.

Previous studies of Hsps have shown that adult females have a greater tolerance than adult males to high temperature stress (Dahlgaard et al., 1998; Sørensen et al., 1999, 2005; Lansing et al., 2000; Andersen et al., 2006; Sarup et al., 2006; Lü and Wan, 2011). *Drosophilia melanogaster* (Dahlgaard et al., 1998; Lansing et al., 2000) females can tolerate high temperature stress better than males, and this is also the case for *Drosophila buzzatii* (Sørensen et al., 1999, 2005; Sarup et al., 2006) and *Aedes aegypti* (Andersen et al., 2006). In our study, whether adult females have a greater tolerance than adult males to high temperature stress needs to be further investigated. Knockdown of one of the three *Hsp* (*Hsp70*, *Hsp21*, and *sHsp21*) genes in *A. hygrophila* females reduced their reproduction significantly, and silencing of these *Hsp* genes also affected adult survival. Therefore, we speculated that *Hsp70*, *Hsp21*, and *sHsp21* genes play important roles in the thermo-tolerance of *A. hygrophila*.

## Conclusion

We obtained complete cDNA of three *Hsps* encoding the ORFs of *A. hygrophila* and analyzed the amino acid sequences and gene structures. The phylogenetic tree showed that *Hsp70*, *Hsp21*, and *sHsp21* were relatively conservative in evolution, and their *Hsps* were highly homologous to those of Coleoptera insects. The expression of three *Hsps* genes was significantly up-regulated after heat shock. RNAi bioassays showed that three *Hsps* played important roles in *A. hygrophila* heat resistance and fecundity. The present results could improve our understanding of the mechanisms of thermotolerance in *A. hygrophila* at the molecular level.

## Data Availability Statement

The datasets generated for this study can be found in the GenBank (accession numbers: MN138034 and MN163037–MN163038).

## Author Contributions

JJ, JG, and ZZ conceived and designed the experiments. JJ and JG performed the experiments and wrote the manuscript. JJ, MZ, and ZZ analyzed the data. YW and FW provided the technical support.

## Conflict of Interest

The authors declare that the research was conducted in the absence of any commercial or financial relationships that could be construed as a potential conflict of interest.

## Footnotes

1 https://blast.ncbi.nlm.nih.gov/Blast.cgi

2 https://www.ncbi.nlm.nih.gov/orffinder/
