# Supplementary material for: Induced Thermotolerance and Expression of Three Key Hsp Genes (Hsp70, Hsp21, and sHsp21) and Their Roles in the High Temperature Tolerance of Agasicles hygrophila
Source: Front Physiol. 2020 Jan 14;10:1593. doi: 10.3389/fphys.2019.01593 (PMC6971057; doi:10.3389/fphys.2019.01593)
Supplement: Supplementary file 1 [file Data_Sheet_1.doc]

**Supplementary Materials:**

**Supplementary Table S1 Gene sequences of PCR primers for three heat shock proteins (*Hsp70*, *Hsp21*, and *sHsp21*)**

| **Primer name** | **Primer sequence** | **Product lenght（bp）** |
| --- | --- | --- |
| primer-1-F | TGATGTTGTTCTTGTTGGTGGTTC | 304 |
| primer-1-R | ATCTGCGTATGTTGTAAATGTCTG |  |
| q2-70-F | GCCACAGCTGGTGACACACATCT | 137 |
| q2-70-R | AGCTCTTTCGGCAGCAGTCCTT |  |
| R-5’-outer | CTGTGGCTTATGGAGCTGCCGTTCA | 932 |
| R-5’-innerer | CACTGGGAATTGAGACAGCAGGAGG | 835 |
| R-3’-innerer | CTCCTCCTGCTGTCTCAATTCCAGTG | 806 |
| R-3’-outer | TGGTCATAACCCCTCCTGCTGTCT | 841 |
| ds-591,894-F | TGATGTTGTTCTTGTTGGTGGTTC | 298 |
| ds-591,894-R | ATCTGCGTATGTTGTAAATGTCTG |  |
| Hsp21-3-F | GGCTTTGTTCAGACATTTATT | 329 |
| Hsp21-3-R | TCTTTCTTCGTGTTTACCTTC |  |
| q2-21-F | GGACTTCGTTTTACCCTTACTTGC | 167 |
| q2-21-R | CTTCAGGATGGAAATGTTGGAC |  |
| R-5’-outer | TCCACAGATGGTGTGCTGTCAGTCA | 418 |
| R-5’-innerer | TGTCAGTCACAGCTCCCAGAAACG | 434 |
| R-3’-innerer | CCGTTTCTGGGAGCTGTGACTGA | 458 |
| R-3’-outer | TGTTTGGGTAGCTCCGTTTCTGGG | 470 |
| ds-93,536-F | GGACTTCGTTTTACCCTT | 444 |
| ds-93,536-R | TCTTCCCCTTCTTCTTTC |  |
| sHsp21-3-F | ATTGTCTTCTGATGGGGTGTTGTC | 108 |
| sHsp21-3-R | AGTTTAGCCGGTTCTCCAGTTTGT |  |
| q2-s21-F | TTGTCTTCTGATGGGGTGTTG | 99 |
| q2-s21-R | CGGTTCTCCAGTTTGTGTTATAGG |  |
| 5.3’outer | GCTGTCAACGATACGCTACGTAAC |  |
| 5.3’inner | GCTACGTAACGGCATGACAGTG |  |
| R-sHsp21-F1 | AGCAGTATGATGTTAGTAGAGTTGGATCGAAATT | 386 |
| R-sHsp21-F2 | CGATTACGGCTCCAAGAATTATTGAAGAAG | 440 |
| R-sHsp21-R | GCCGGTTCTCCAGTTTGTGTTATAGG | 518 |
| R-sHsp21-R | CGACAACACCCCATCAGAAGACAA | 441 |
| ds-145,568-F | TATCCATCTGGTTACCTACG | 423 |
| ds-145,568-R | CTTTCTTTTCAATCGACTTC |  |

**
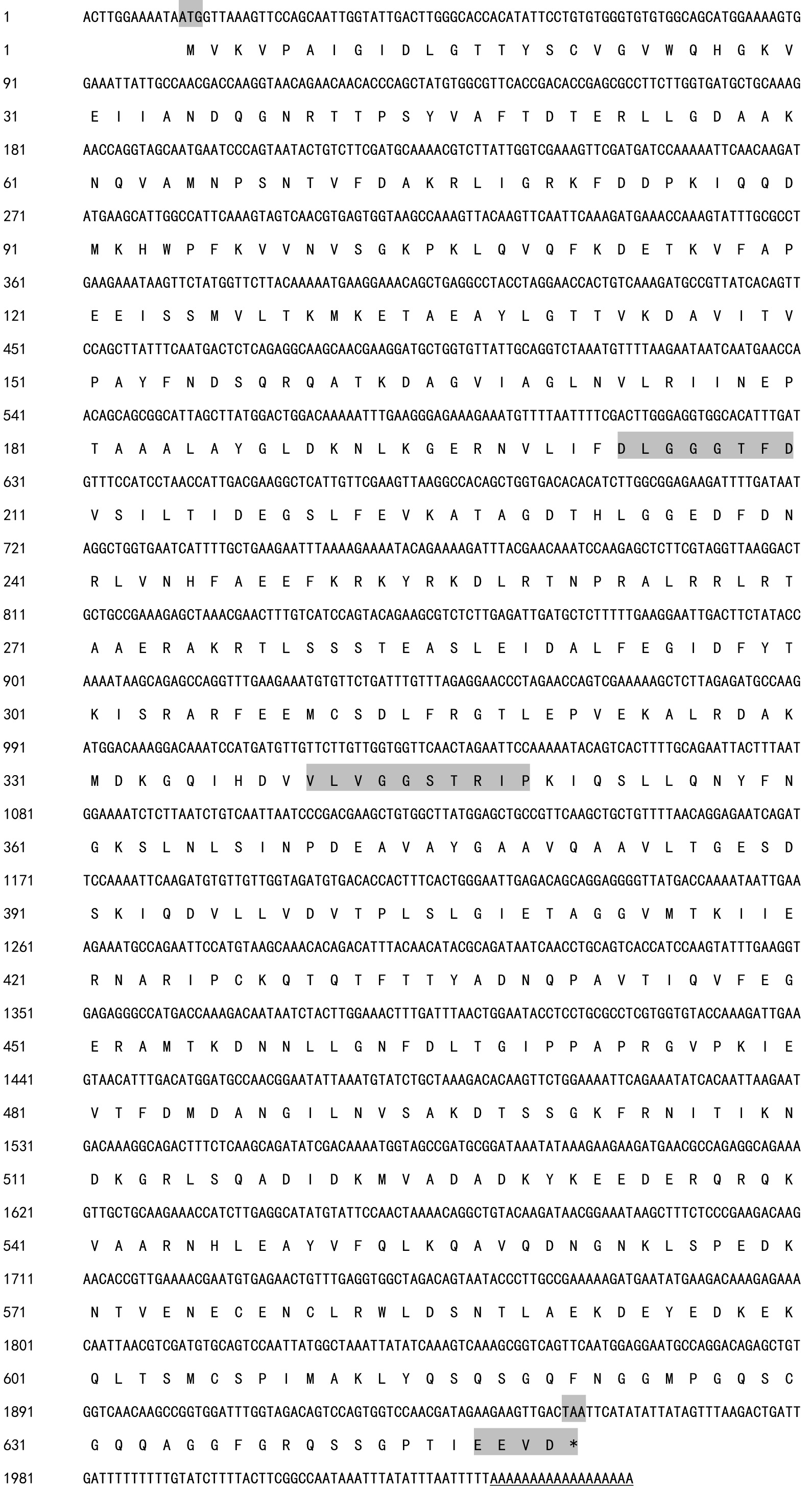
**

**Supplementary Figure** S1: **Nucleotide and deduced amino acid sequences of *Hsp70* cDNA from *A. hygrophila***. The character shading (ATG) indicates the translational start codon. The asterisk indicates the translational termination codon (TAA). The termination signal is in bold, and the poly(A) tail is underlined. The amino acid sequence derived from the cloned gene was analyzed, and the amino acid polypeptide chain contained highly conserved sequences of DLGGGTFD, VLVGGSTR, and GPTIEEVD. The sequence, EEVD, is the characteristic motif of cytoplasmic *Hsp70*.


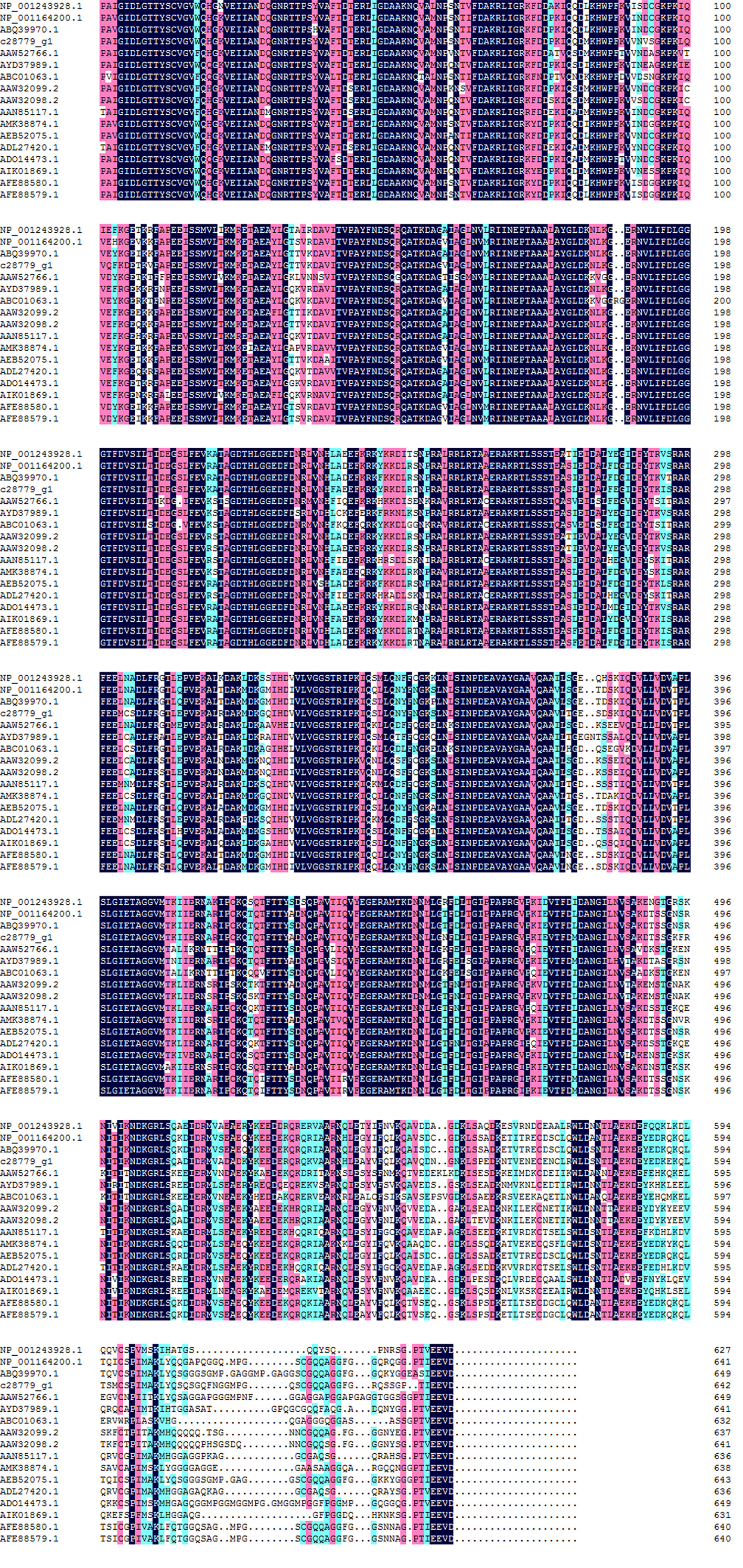


**Supplementary Figure** S2: **Multiple sequence alignment of the amino acid sequences of *Hsp70* in *A. hygrophila* and other insects**

Note: *Bombyx mori* (NP_001243928.1), *Tribolium castaneum* (NP_001164200.1), *Anatolica polita borealis* (ABQ39970.1), *Mytilus galloprovincialis* (AAW52766.1), *Trichogramma chilonis* (AYD37989.1), *Procambarus clarkii* (ABC01063.1), *Liriomyza sativae* (AAW32099.2), *Liriomyza huidobrensis* (AAW32098.2), *Chironomus tentans* (AAN85117.1), *Colaphellus bowringi* (AMK38874.1), *Microdera punctipennis* (AEB52075.1), *Chironomus riparius* (ADL27420.1), *Bemisia tabaci* (ADO14473.1), *Orius sauteri* (AIK01869.1), *Tenebrio molitor* (AFE88580.1), *Tenebrio molitor* (AFE88579.1).


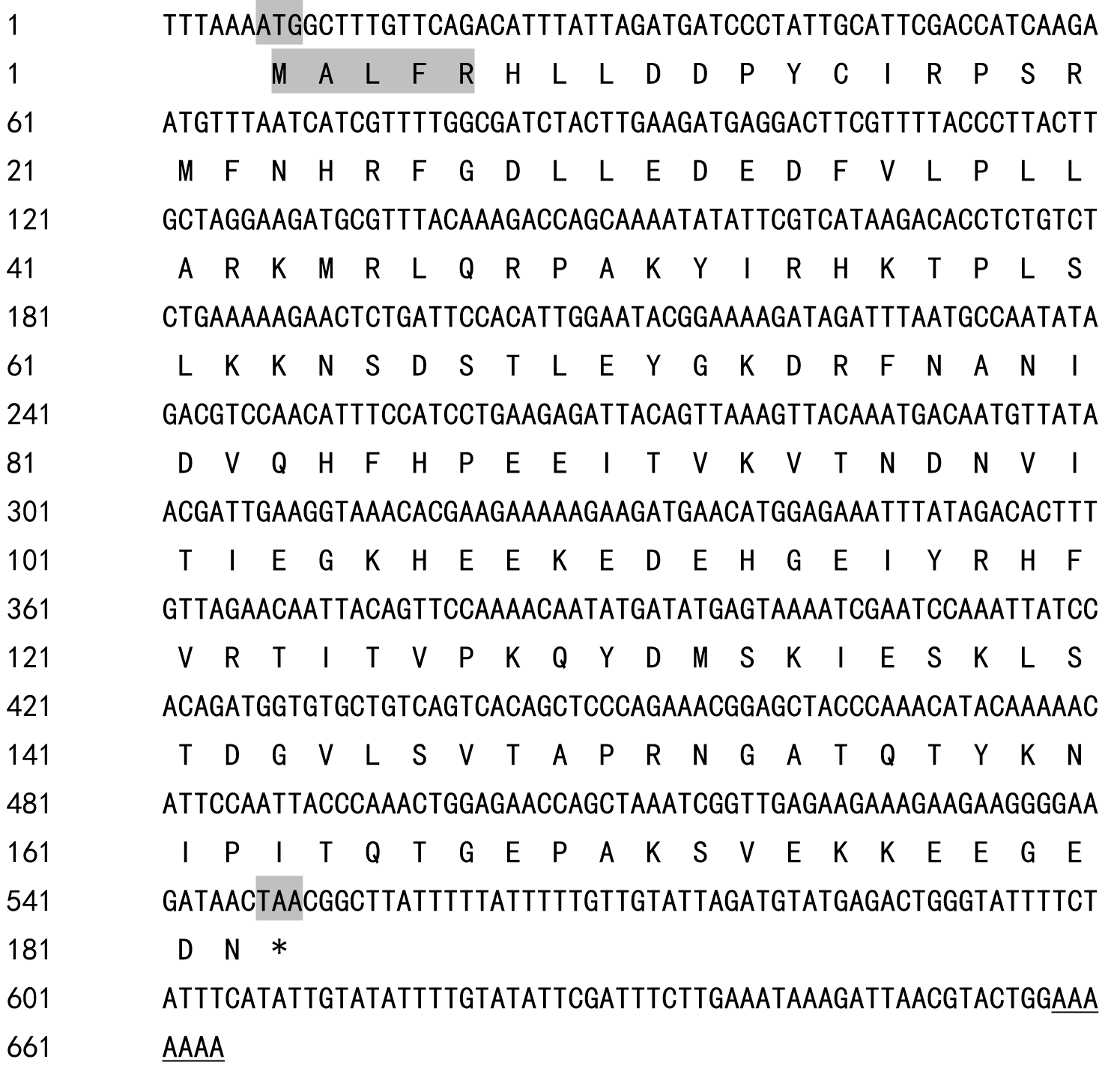


**Supplementary Figure** S3: **Full-length cDNA sequence of *Hsp21* and its deduced amino acid sequence.** The character shading (ATG) indicates the translational start codon. The asterisk indicates the translational termination codon (TAA). The highly conserved sequence, MALFR, is a typical sequence characteristic of *Hsp21* among other insects.


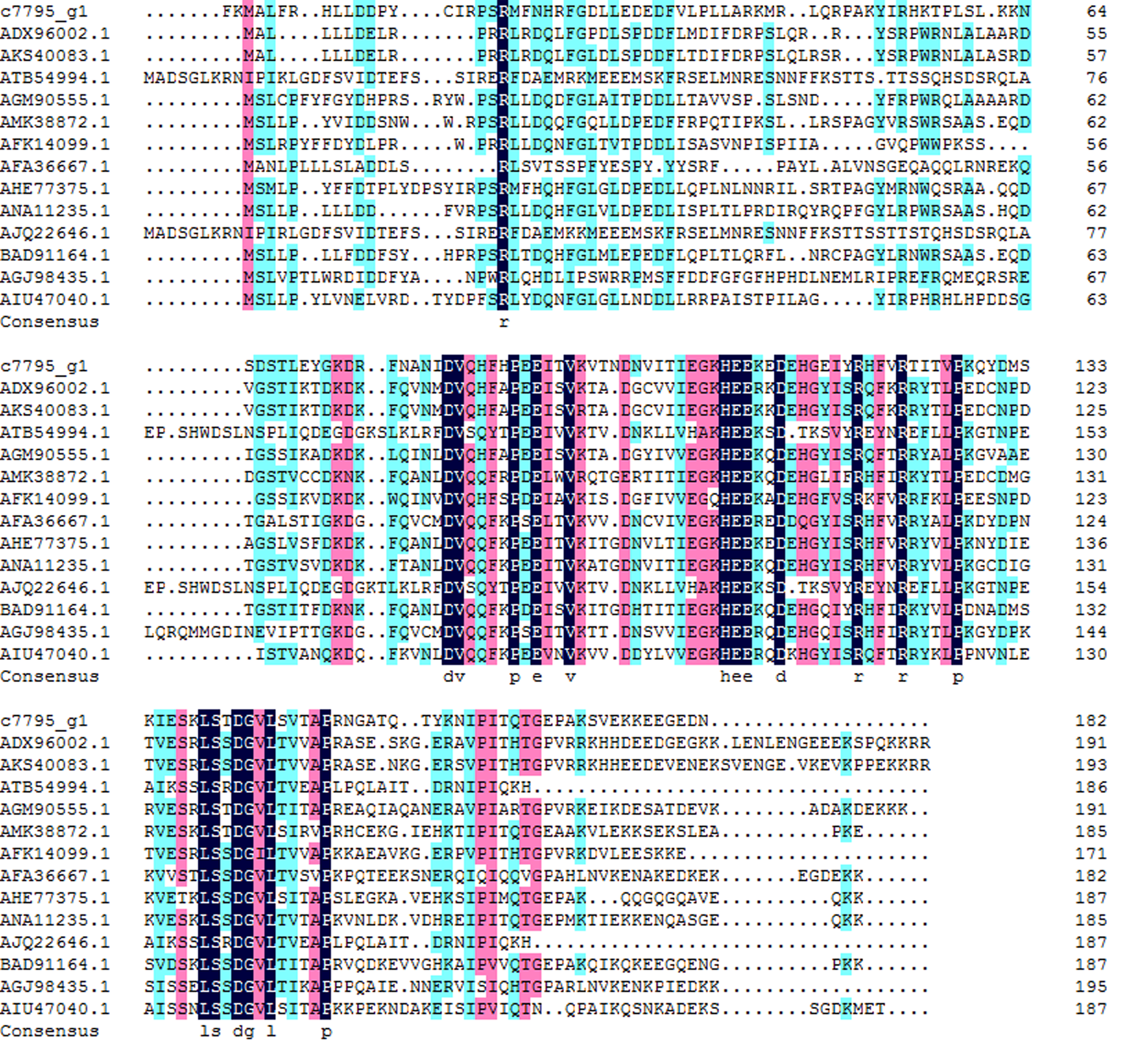


**Supplementary Figure** S4: **Multiple sequence alignment of the amino acid sequences of *Hsp21* in *A. hygrophila* and other insects.**

Note: *Helicoverpa armigera* (ATB54994.1), *Chilo suppressalis* (AGM90555.1), *Lissorhoptrus oryzophilus* (AHE77375.1), *Dastarcus helophoroides* (ANA11235.1), *Antheraea pernyi* (AJQ22646.1), *Gastrophysa atrocyanea* (BAD91164.1), *Chironomus riparius* (AGJ98435.1), *Phenacoccus solenopsis* (AIU47040.1), *Colaphellus bowringi* (AMK38872.1), *Spodoptera litura* (AFK14099.1), *Lucilia cuprina* (AFA36667.1), *Cydia pomonella* (ADX96002.1), *Grapholita molesta* (AKS40083.1).


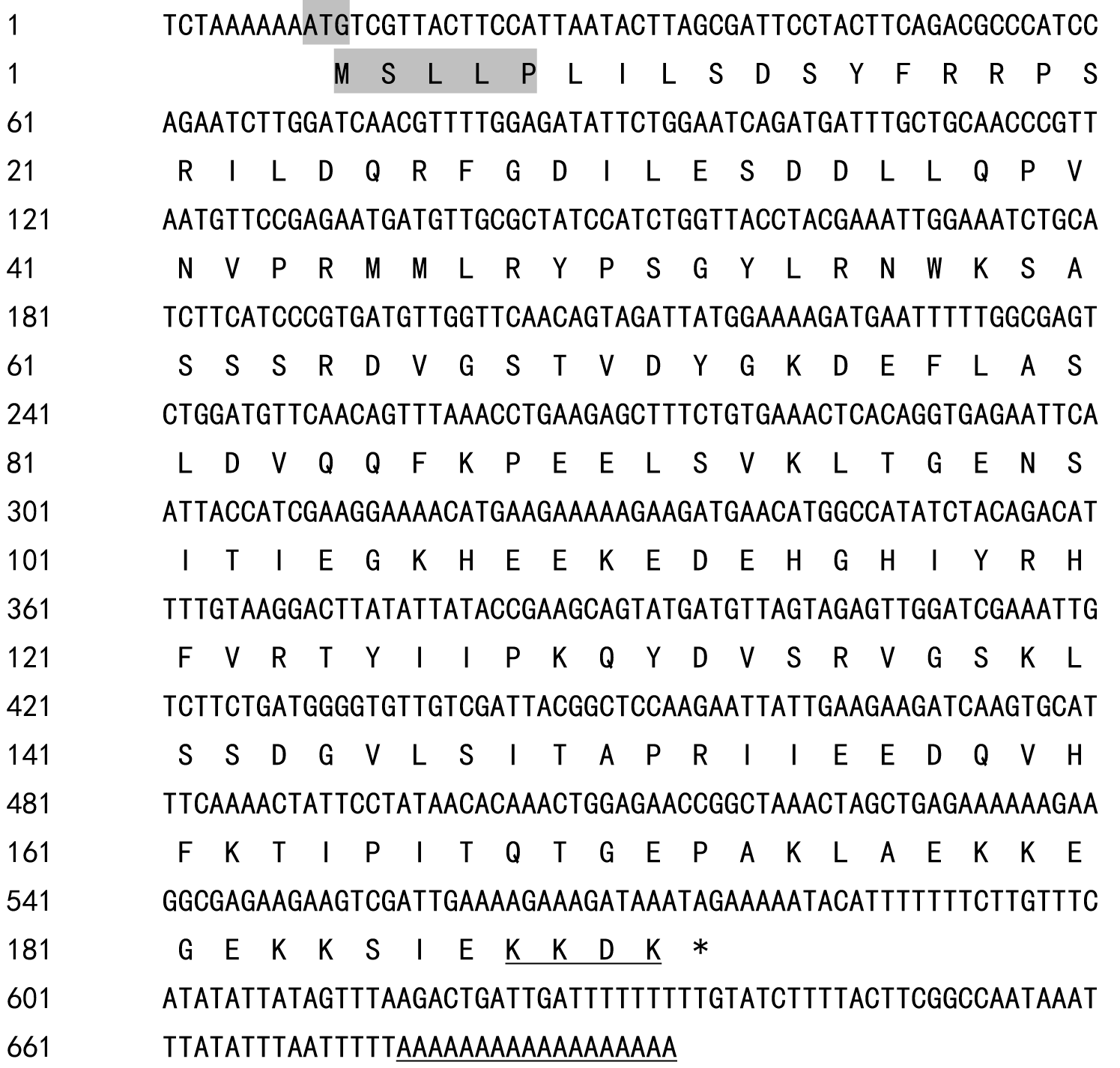


**Supplementary Figure** S5: **Nucleotide and deduced amino acid sequences of *sHsp21* cDNA from *A. hygrophila*.** The character shading (ATG) indicates the translational start codon. The asterisk indicates the translational termination codon (TAA). The highly conserved sequence, MSLLP, was a typical sequence characteristic of *sHsp21* in other insects.


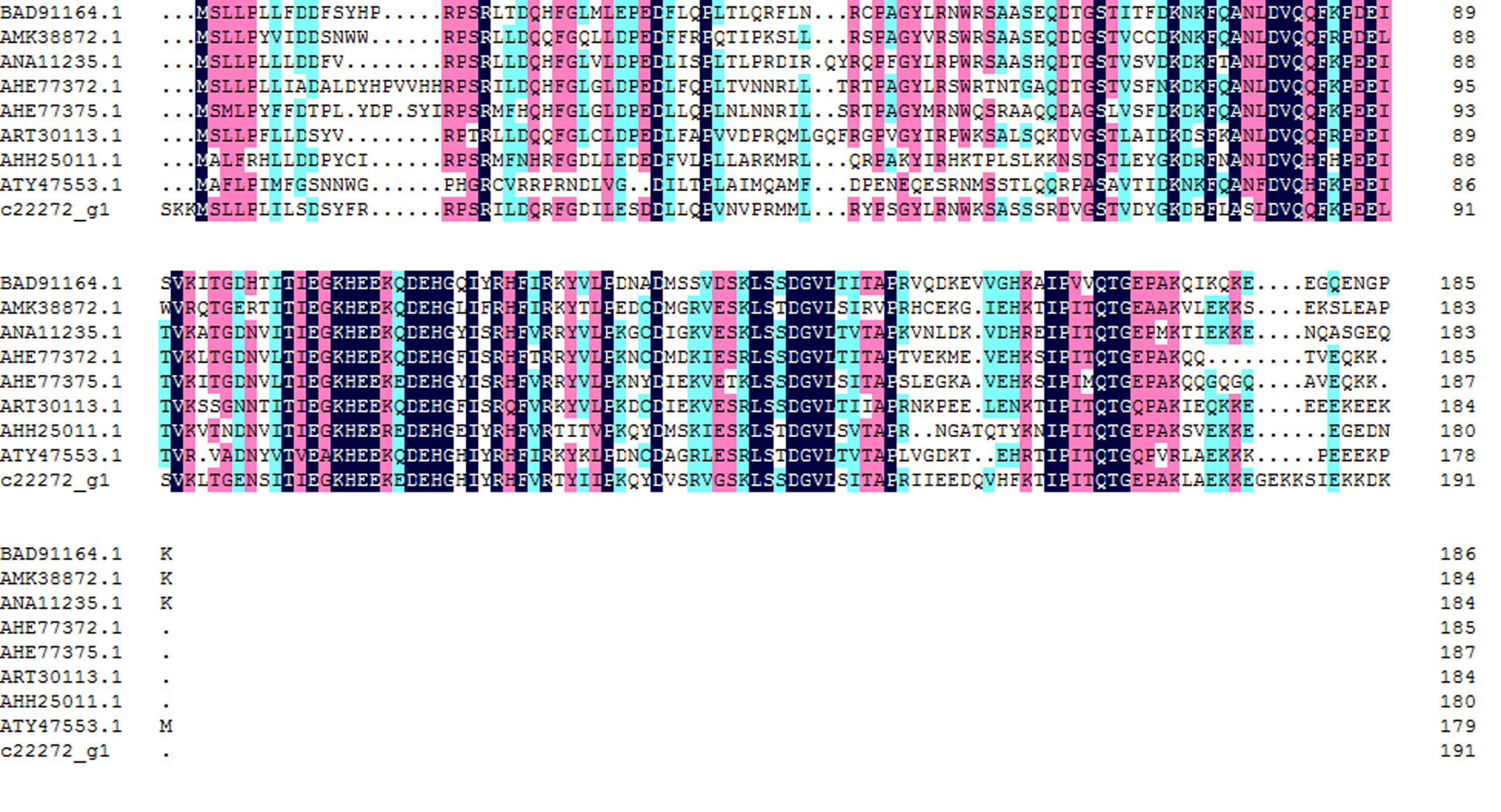


**Supplementary Figure** S6: **Multiple sequence alignment of the amino acid sequences of *sHsp21* in *A. hygrophila* and other insects.**

Note: *Gastrophysa atrocyanea* (BAD91164.1), *Colaphellus bowringi* (AMK38872.1), *Dastarcus helophoroides* (ANA11235.1), *Lissorhoptrus oryzophilus* (AHE77372.1), *Lissorhoptrus oryzophilus* (AHE77375.1), *Harmonia axyridis* (ART30113.1), *Agasicles hygrophila* (AHH25011.1), *Galeruca daurica* (ATY47553.1).
